# Targeting Angiogenesis in Squamous Cell Carcinoma of the Head and Neck: Opportunities in the Immunotherapy Era

**DOI:** 10.3390/cancers14051202

**Published:** 2022-02-25

**Authors:** Nabil F. Saba, Pooja Vijayvargiya, Jan B. Vermorken, Juan P. Rodrigo, Stefan M. Willems, Nina Zidar, Remco de Bree, Antti Mäkitie, Greg T. Wolf, Athanassios Argiris, Yong Teng, Alfio Ferlito

**Affiliations:** 1 Department of Hematology and Medical Oncology, Winship Cancer Institute, Emory University, Atlanta, GA 30322, USA; pvijayv@emory.edu (P.V.); yong.teng@emory.edu (Y.T.); 2 Department of Medical Oncology, Antwerp University Hospital, 2650 Edegem, Belgium; janb.vermorken@uza.be; 3 Faculty of Medicine and Health Sciences, University of Antwerp, 2650 Antwerp, Belgium; 4 Department of Otolaryngology, Hospital Universitario Central de Asturias, University of Oviedo, ISPA, IUOPA, CIBERONC, 33011 Oviedo, Spain; jprodrigo@uniovi.es; 5 Department of Pathology and Medical Biology, University Medical Center Groningen, 9727 GZ Groningen, The Netherlands; swille18@umcutrecht.nl; 6 Faculty of Medicine, Institute of Pathology, University of Ljubljana, Korytkova 2, 1000 Ljubljana, Slovenia; nina.zidar@mf.uni-lj.si; 7 Department of Head and Neck Surgical Oncology, University Medical Center Utrecht, 3584 CX Utrecht, The Netherlands; r.debree@umcutrecht.nl; 8 Department of Otorhinolaryngology—Head and Neck Surgery, HUS Helsinki University Hospital, University of Helsinki, FI-00029 Helsinki, Finland; antti.makitie@hus.fi; 9 Department of Otolaryngology—Head and Neck Surgery, University of Michigan Medical School, Ann Arbor, MI 48109, USA; gregwolf@med.umich.edu; 10 Department of Medical Oncology, Thomas Jefferson University, Philadelphia, PA 19107, USA; athanassios.argiris@gmail.com; 11 International Head and Neck Scientific Group, 35100 Padua, Italy; a.ferlito@uniud.it

**Keywords:** angiogenesis, immunotherapy, VEGF, SCCHN, tumor microenvironment

## Abstract

**Simple Summary:**

Therapies for squamous cell carcinomas of the head and neck (SCCHN) have been rapidly evolving, initially with the inclusion of immunotherapy, but more recently with the consideration of anti-angiogenic therapies. Recent preclinical and clinical data reveal a strong correlation between vascular endothelial growth factor (VEGF) and the progression of SCCHN, with nearly 90% of these malignancies expressing VEGF. Our review article not only elaborates on the utility of anti-VEGF therapies on SCCHN but also its interaction with the immune environment. Furthermore, we detailed the current data on immunotherapies targeting SCCHN and how this could be coupled with anti-angiogenics therapies.

**Abstract:**

Despite the lack of approved anti-angiogenic therapies in squamous cell carcinoma of the head and neck (SCCHN), preclinical and more recent clinical evidence support the role of targeting the vascular endothelial growth factor (VEGF) in this disease. Targeting VEGF has gained even greater interest following the recent evidence supporting the role of immunotherapy in the management of advanced SCCHN. Preclinical evidence strongly suggests that VEGF plays a role in promoting the growth and progression of SCCHN, and clinical evidence exists as to the value of combining this strategy with immunotherapeutic agents. Close to 90% of SCCHNs express VEGF, which has been correlated with a worse clinical prognosis and an increased resistance to chemotherapeutic agents. As immunotherapy is currently at the forefront of the management of advanced SCCHN, revisiting the rationale for targeting angiogenesis in this disease has become an even more attractive proposition.

## 1. Clinical Evidence for Targeting VEGF in SCCHN

Anti-angiogenic agents have gained significant importance as therapeutic options for various malignancies [1,2,3]. Biologically, tumor proliferation and growth depend on nutrient and blood delivery, mediated through new vessel formation, which is the process of angiogenesis [4]. Increased vascular density has been reported to be associated with tumor progression and metastases [2,5]. Therefore, therapies targeting pro-angiogenic factors have been a focus of interest in oncology over the past 2 decades [6,7].

Angiogenesis is a multi-step process involving the protease breakdown of basement membrane allowing for the migration and proliferation of endothelial cells, leading to the formation of a new lumen with a basement membrane, pericytes, a remodeled extracellular matrix, and ultimately anastomoses with blood flow [8]. These intricate processes and their inhibition likely play a major role in impacting the tumor microenvironment where immune cells often reside. In addition, tumor cells heavily depend on this mechanism for their own development and are unable to expand past 2–3 mm^3^ given diffusion-dependent resources [5]. Since both immune-mediated factors and those that promote or inhibit angiogenesis coexist in the tumor microenvironment, exploring possible anti-tumor synergistic mechanisms targeting these two cancer-related processes (immunity and angiogenesis) seems attractive.

Angiogenesis is largely instigated by the activation of tyrosine kinase receptors, notably vascular endothelial growth factor (VEGF), epidermal growth factor (EGF), platelet derived growth factor (PDGF), and fibroblast growth factor (FGF) [1,9,10,11]. The upregulation of these angiogenic factors typically corresponds to increased vascularity, lymph node metastasis, inadequate response to cytotoxic chemotherapy, and advanced disease with poor prognosis [1,10]. Up to 90% of SCCHNs have been shown to express VEGF which promotes immunosuppression in different ways, namely by reducing T-cell extravasation across vessel walls, enhancing regulatory T-cell differentiation, stimulating dendritic PD-L1 expression which decreases T-cell activation, and finally, by directly inhibiting the differentiation of myeloid stem cells to mature immune regulators by binding their VEGF receptor 1 [4]. This ultimately raises the question of VEGF’s role in tumorigenesis and its possible influence on prognosis in SCCHN.

Several observational reports have attempted to correlate VEGF with clinical or pathologic findings in SCCHN. Tanigaki et al. examined the expression of VEGF-A and -C, and their receptors, Flt-1 and Flt-4, in biopsy specimens taken from 73 patients with tongue carcinoma by immunohistochemistry [12]. Multivariate analyses revealed VEGF-C expression to be an independent factor predicting lymph node metastasis [12]. There were notable differences between VEGF-C-positive and VEGF-C-negative cases in terms of predicting 5-year overall survival (51.7% vs. 94.2%, respectively) [12]. Notably, the 5-year survival rates for VEGF-C-positive and negative patients were 94% and 52%, respectively [12]. Cheng et al. similarly applied immunohistochemistry to examine the expression of VEGF in 100 specimens of oral cavity carcinomas, including 66 oral epithelial dysplasia and 36 normal mucosae [13]. There was a gradual increase in VEGF through the different dysplasia grades from normal mucosa to invasive carcinoma, indicating that VEGF expression is at least a possible predictor of tumor progression [13]. They also showed a correlation between VEGF levels and lymph node metastases (*p* = 0.022) as well as worse survival (*p* = 0.016) and advanced clinical stage (*p* = 0.046) [13]. In a similar fashion, Seibold et al. investigated VEGF and its receptor tyrosine kinase 1 (FLT-1) in patients with locally advanced squamous cell carcinoma who had been treated with adjuvant radiotherapy or chemo-radiotherapy, which showed a correlation between VEGF expression and loco-regional control (LRC), metastasis-free survival, and overall survival (OS) [14]. However, other studies have shown mixed results in terms of outcomes and survival. One 30-patient study of patients with laryngeal cancer showed an association of VEGF expression with lymph node involvement but not with treatment outcomes [15]. Similarly, a 40-patient analysis of SCCHN correlated VEGF expression with staging, but no statistically significant connection existed with disease-free survival or OS [16]. Notably, a meta-analysis evaluating five different biomarkers, including VEGF, in oral tongue squamous cell carcinoma with regard to their prognostic significance on OS yielded insufficient and inconclusive results [17].

There are four general categories of anti-angiogenic agents: ligand-directed antibodies, receptor-directed antibodies, small molecule inhibitors, and immunomodulatory agents [4]. While there are no Food and Drug Administration (FDA)-approved anti-angiogenic agents for SCCHN, several studies have used VEGF and VEGFR inhibitors in the treatment of SCCHN. While certain tyrosine kinase inhibitors (TKIs) have shown some activity against angiogenesis in preclinical studies, this did not consistently translate into meaningful clinical activity in SCCHN. Sorafenib and sunitinib are TKIs with activity against multiple receptors and have shown moderate response to SCCHN in phase II trials [18,19,20,21]. However, many adverse side effects, most commonly fatigue (32%) and grade 3–5 bleeding (16%), were commonly seen with sunitinib [20,21]. Axitinib was studied in a phase II trial with an overall low response rate (6.7%) but an encouraging disease-control rate of 77% and an OS of 10.9 months [22].

It is important to clarify that while anti-angiogenic agents can treat malignancy, they have rarely been associated with curative potential as single agents. A combinatorial approach with cytotoxic therapy has yielded improved responses and disease control with these agents [23]. A combination approach with chemotherapy has been tested in advanced SCCHN in a phase III clinical trial E1305 comparing platinum therapy (cisplatin or carboplatin) plus either docetaxel or 5-fluorouracil (5-FU) with or without bevacizumab for patients with recurrent or metastatic SCCHN [24]. In this 403-patient cohort, the addition of bevacizumab led to an improved median progression-free survival (PFS) from 4.3 months to 6.0 months (HR 0.71; *p* = 0.0012) and an improved overall response rate (ORR) from 24.5 to 35.5% (*p* = 0.013) [24]. However, the median OS was 12.6 months with chemotherapy + bevacizumab versus 11 months with chemotherapy alone, without a statistically significant difference (HR 0.87, 95% CI 0.70–1.0, *p* = 0.22), but with higher observed treatment-associated toxicities in the bevacizumab arm, most notably grade 3–5 bleeding [24]. Despite the fact that the study did not meet its primary endpoint, it did show the clinical activity of anti-angiogenesis in SCCHN, namely in its ability to prolong PFS, and opened the door for further investigation of this approach. Something to note is that E1305 preceded the era of immunotherapy. Anti-angiogenic agents have also been investigated in combination with radiotherapy and epidermal growth factor inhibitors such as cetuximab [4,25,26,27].

## 2. The Immune Correlation with Anti-Angiogenesis

Before investigating the effects of angiogenesis inhibition on the immune system, we must consider the consequences that powerful angiogenic regulators such as VEGF have in order to create an immunosuppressive environment by downregulating immune effector cells [9].

An example is the effect on natural killer cells (NKs), where VEGF causes reduced NK cytotoxicity leading to immunosuppression [28]. VEGF also inhibits dendritic cell (DC) maturation [9], notably by binding with VEGFR-2, attaching to the surface of DCs, and directly impeding nuclear factor-kB signaling [29]. Aside from preventing DC differentiation, VEGF obstructs DCs from presenting antigens to T cells by upregulating programmed death ligand-1 (PD-L1) expression, which in turn exerts an effect on T-cell activation [30]. Consequentially, VEGF blockade relieves the restrictions on DC migration and immune capacity via increased antigen presentation and may be a promoter of anti-tumor immunity [31]. In mouse models with glioblastoma, anti-VEGF resulted in the increased co-stimulatory expression of B7-1, B7-2, and MHC class II molecules, creating more advanced dendritic cell identity [32].

Similarly, pro-angiogenic molecules directly act on T lymphocytes by binding with VEGFR-2 and upregulating immune checkpoints such as PD-1 and cytotoxic T-lymphocyte-associated protein 4 (CTLA-4) [33]. This ultimately leads to the upregulation of regulatory T cells (Tregs) [33]. VEGF has also been shown to act directly on T lymphocytes, with the most notable effect being its inhibition of hematopoietic stem cell differentiation to CD8+ and CD4+ T cells [34,35,36]. This was effective in causing T-cell deficiency and atrophy of the thymus when examined in cancer patients and animal models with tumors [34]. In oral squamous cell carcinoma specifically, VEGF has been shown to enhance the secretion of prostaglandin E2, which interrupts T-cell activation [37]. Besides preventing T-cell adhesion to vessel wall and subsequent extravasation to the tumor site [30], VEGF also inhibits helper T-cell recruitment to the tumor site [38] and promotes immunosuppressive cells such as Tregs [30,39] and myeloid-derived suppressor cells (MDSCs) [40] by binding with VEGFR-2. It also achieves this upregulation of Tregs by combining with the co-receptor neuropilin 1 [41].

Pro-angiogenic molecules repress adhesion factors and chemokines such as CXC chemokine ligands 10/11, vascular cell adhesion molecule-1 (VCAM-1), intracellular adhesion molecule-1 (ICAM-1), and endothelial leukocyte adhesion molecule 1 (ELAM-1), which would normally attract NK and CD8+ T cells [42,43]. Therefore, anti-angiogenesis improves T-cell infiltration of the tumor environment by upregulating adhesion molecules on nearby vessels [44]. Overall, anti-angiogenic therapy therefore reprograms the microenvironment, favoring an upregulation of immunomodulators and more potent anti-tumor response. These findings provide sufficient evidence to support the hypothesis that the co-targeting of tumor-mediated angiogenesis through VEGF with pro-tumor-mediated immune factors may be a winning strategy in anti-cancer care, particularly in SCCHN, which relies on both elements.

## 3. The Prospects of Combination of VEGF Inhibitors with Immunotherapy in SCCHN

Immunotherapy has surfaced as a breakthrough in cancer treatment, notably for patients with recurrent or metastatic SCCHN [45,46]. Tumors typically express different immune checkpoint receptors as a means for immune evasion. By targeting these receptors, cancer immune evasion is reversed. In metastatic SCCHN, prior to the integration of immunotherapy, first-line systemic therapies consisted of a combination of cytotoxic agents with cetuximab, a chimeric IgG1 monoclonal antibody targeting human EGFR [25,47]. Even though the introduction of immune checkpoint inhibitors (ICIs) has revolutionized the treatment of recurrent or metastatic disease [48], most patients still succumb to their disease, and novel therapeutic combinatorial approaches are urgently needed.

**Figure 1:**
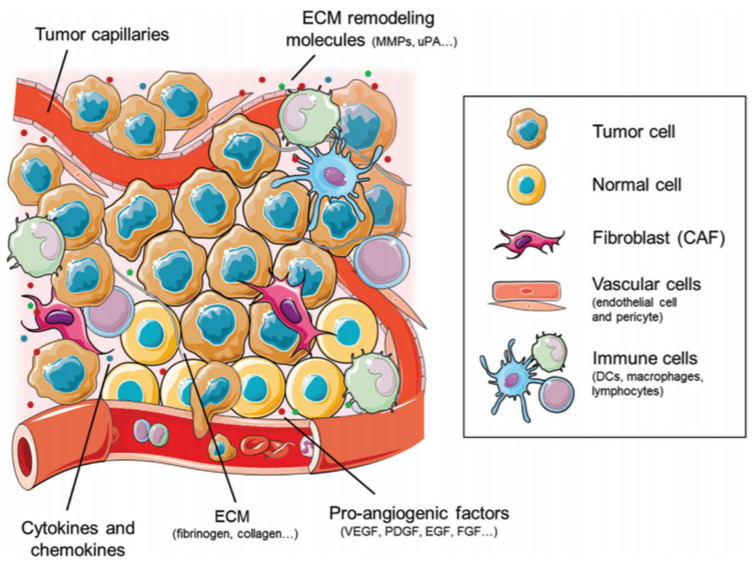
A closer look at the cellular and molecular components of the tumor microenvironment from the immune cells and chemokines to the extracellular matrix and their associated remodeling molecules that shape the interplay between tumor cells and host immune cells along with pro-angiogenic factors, highlighting the potential targets for therapy [49].

**Figure 2:**
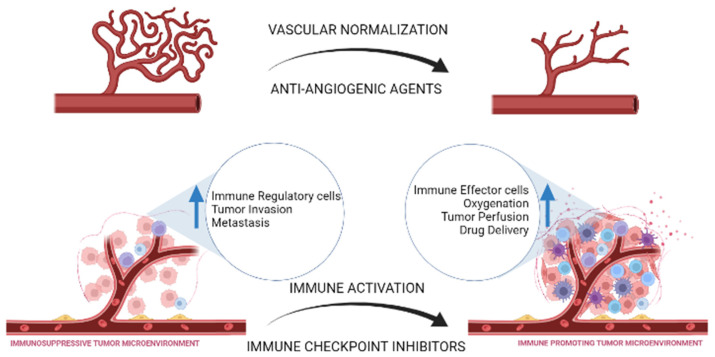
Highlighting the parallel between anti-angiogenic agents and immune checkpoint inhibitors in their effect on the tumor microenvironment and how it impacts tumor progression and drug delivery. Created with biorender.com (accessed on 16 February 2022).

The tumor microenvironment (TME) is an essential factor for tumor survival and growth, showcasing the importance of therapies that threaten this environment [9]. Closely regulated by immune and inflammatory cells, cytokines, and the surrounding tissue and vessels as illustrated in Figure 1 [49], the TME is promoted by increased vascularity, resistance to host immune cells, and the ability to combat hypoxia [50,51]. Therefore, therapies targeting both the immune system and angiogenesis are appealing. These novel approaches may help promote the normalization of vasculature and an immune boosting rather than a suppressive environment [9], as delineated in Figure 2 [44].

There are multiple reasons for the success of this dual-modality approach. First, tumor cells actively promote angiogenic factors, which not only stimulate abnormal vascular structure but also certain chemokines and adhesion molecules, which selectively impede the infiltration of immune cells [52]. This setting restricts the effectiveness of immunotherapy [52]. Second, existing therapies for SCCHN that target one driver, such as ICIs and anti-angiogenic agents, may be limited when tumors paradoxically utilize other pro-tumor mediators that can counteract their efficacy [45,53]. For example, PD-L1 inhibition consequently increases the expression of other immune checkpoints such as TIM-3, potentially causing an adaptive resistance [54,55]. Similarly, anti-VEGF treatments generate hypoxia and acidosis via abnormal vessel pruning [45]. This environment leads to an increased expression of CCL28 and SDF-1, which induces an immunosuppressed TME by promoting tolerance in T regulatory cells, MDSCs, and TAMs [56,57]. TME hypoxia also compromises antigen-presenting cells and subsequent activation of T-cell response. This not only impacts the efficacy of anti-cancer agents but also leads to the dysfunction of immune effector cells and the recruitment of tumor-enhancing cells [58,59,60,61].

Given the close interaction between angiogenic factors and immune response, the combination of anti-angiogenic therapy and ICIs has become an attractive strategy to combat the resistance mechanisms that tumor cells utilize to evade the effects of therapy. By improving the penetration of concurrent therapies, targeting both the TME and the tumor itself, and by helping build resistance to immune-evading tumor strategies, anti-angiogenic therapy and immunotherapy provide a promising opportunity for the future of SCCHN therapy. Numerous active clinical trials exist to assess this presumed synergistic effect, as noted in Table 1.

NCT03650764 is a prospective phase I/II trial studying pembrolizumab with ramucirumab, a VEGF inhibitor, in recurrent/metastatic SCCHN [62]. Another similar phase II clinical trial, NCT04440917, is testing another PD-1 inhibitor, camrelizumab, with VEGFR inhibitor apatinib in locally advanced SCCHN [63].

A phase II clinical trial, NCT03468218, is evaluating the combination of pembrolizumab and cabozantinib (a TKI targeting multiple receptors, including VEGFR2) in recurrent or metastatic SCCHN [64]. While the results have not yet been published, we are encouraged by the clinical trial results in renal cell carcinoma comparing nivolumab plus cabozantinib with sunitinib monotherapy. In this study, the median PFS for nivolumab plus cabozantinib was 16.6 months (95% CI, 12.5–24.9), while for sunitinib, it was 8.3 months (95% CI, 7.0–9.7); this trend was consistently seen for OS and objective response rate (ORR) [65].

Multiple other studies have examined the utility of bevacizumab in combination with immune and/or chemotherapy. NCT03818061 is a phase II multicenter study assessing the effects of atezolizumab (a PD-L1 inhibitor) and bevacizumab in recurrent or metastatic SCCHN on ORR [66]. Other trials compare bevacizumab with cetuximab, which may not only work through receptor blockade but also via the immune-mediated activity of cetuximab [67]. A phase II trial, NCT00409565, evaluated the ORR of bevacizumab with cetuximab in patients with recurrent or metastatic SCCHN [68]. Published results revealed a significant reduction in tumor vascularization, with an ORR of 16%, a disease control rate of 73%, and a generally well-tolerated response with grade 3–4 adverse events in less than 10% [69]. Three specific phase II trials are evaluating bevacizumab + cetuximab +/− chemoradiation: NCT00968435 with a combination of bevacizumab, cisplatin, cetuximab, and intensity-modulated radiation therapy (IMRT) to determine 2-year PFS for locally or regionally advanced SCCHN [70], NCT00703976 evaluating bevacizumab, cetuximab, pemetrexed, and radiation therapy (RT) for similar outcome and disease population [71], and NCT01588431 with combination induction therapy with bevacizumab, cetuximab, and chemotherapy (docetaxel, cisplatin) followed by radiation, cisplatin, cetuximab, and bevacizumab +/− surgery depending on response [72]. Other trials, including the phase Ib/II trial NCT0250109673, are assessing lenvatinib (a multi-kinase inhibitor including VEGFR) in combination with pembrolizumab in a host of solid tumors, including SCCHN both in the first-line as well as in post-immunotherapy failure.

Along with assessing the potential benefits of combination therapy, we must consider the associated toxicities of anti-angiogenic therapy, which range from cardiovascular to thromboembolic [73]. Some known side effects include hypertension with associated proteinuria and reversible posterior leukoencephalopathy, endocrine dysfunction, and gastrointestinal perforation [74]. Anti-VEGF/VEGFR agents are also associated with thromboembolism in 5% of cases as well as with hemorrhage in others [73]. As these agents are largely TKIs, some effects are secondary to off-target tyrosine kinase inhibition, namely hypothyroidism, diarrhea, and fatigue [75]. A meta-analysis published by Ranpura et al. detailed the increased risk of adverse events associated with bevacizumab compared with chemotherapy alone in regard to mortality (2.9% vs. 2.2%; RR = 1.33; 95% CI 1.02–1.73) and fatal events (3.3% vs. 1%; RR = 3.49; 95% CI 1.82–6.66) [76]. The most common fatal events were noted as bleeding (23.5%), gastrointestinal perforation (7.1%), and neutropenia (12.2%), without a correlation between mortality and the type of cancer or bevacizumab dose [76,77].

## 4. Conclusions

While the use of combination therapy forms an intriguing forefront for the treatment of recurrent and metastatic SCCHN, we have yet to understand how immunotherapy and anti-angiogenic therapy interact with each other to create an anti-tumor effect. Further investigations need to appreciate both the benefits and the risks posed by inhibiting these alternative therapeutic pathways and ultimately how they impact the TME. In conclusion, the approval of immunotherapy as an effective modality in the treatment of SCCHN has ushered a new era in combinatorial therapeutic approaches for this disease. Very high on the list of candidate targeted agents are angiogenesis inhibitors. Here, we attempted to provide a rationale for the need to pursue these combinations in SCCHN. Along those lines, results from the enrolling studies in recurrent metastatic SCCHN are eagerly awaited and may provide more insight into refining these approaches for a wider patient population through better clinical as well as biomarker-based patient selection.

## Figures and Tables

**Table 1:** Ongoing trials combining anti-angiogenic agents with immunotherapy in SCCHN.

Trial Identifier	Phase	Treatment	Tumor Target	Status	Primary Outcome
NCT02501096	Ib/II	Lenvatinib + Pembrolizumab	SCCHN, NSCLC, RCC, EC, UC, Melanoma	Active, not recruiting	MTD, ORR, DLT
NCT03650764	I/II	Ramucirumab + Pembrolizumab	SCCHN, recurrent or metastatic disease	Active, not recruiting	ORR, RP2D of Ramucirumab
NCT03468218	II	Cabozantinib + Pembrolizumab	SCCHN, refractory, recurrent, or metastatic	Recruiting	ORR
NCT03818061	II	Bevacizumab + Atezolizumab	SCCHN, advanced/metastatic	Recruiting	ORR
NCT04428151	II	Lenvatinib + Pembrolizumab vs. SOC chemotherapy and Lenvatinib monotherapy	SCCHN, recurrent or metastatic, first line	Recruiting	ORR
NCT04199104	III	Lenvatinib ± Pembrolizumab	SCCHN, recurrent or metastatic, second line	Recruiting	ORR, PFS, OS

Non-small cell lung cancer (NSCLC); renal cell carcinoma (RCC); endometrial cancer (EC); urothelial cancer (UC); standard of care (SOC); maximum tolerated dose (MTD); overall response rate (ORR); dose-limiting toxicity (DLT); recommended phase 2 dose (RP2D); progression-free survival (PFS); overall survival (OS).
